# Agile Implementation and Expansive Learning: Identifying Contradictions and Their Resolution Using an Activity Theory Perspective

**DOI:** 10.1007/978-3-030-49392-9_1

**Published:** 2020-05-06

**Authors:** Pritam Chita, Peter Cruickshank, Colin Smith, Kendall Richards

**Affiliations:** 6 University of Oslo, Oslo, Norway; 7 Monash University, Clayton, VIC Australia; 8 IT University of Copenhagen, Copenhagen, Denmark; 9 University of British Columbia, Vancouver, BC Canada; Edinburgh Napier University, Edinburgh, UK

**Keywords:** Organisational learning, Activity Theory, Expansive learning, Contradictions, Congruences

## Abstract

A key challenge organisations face when transitioning to agile delivery methods is that of quickly and effectively learning new ways of working. This study posits that fundamental historical, cultural and behavioural aspects affect the transition and contribute to the poor performance of many agile implementations. In order to address such factors, this study applies a modified Activity Theory (AT) based framework to a case study agile implementation within a large public sector organisation. An activity is closely defined, and six generic activities associated with all agile implementations are identified. These are validated against the agile maturity model literature and a set of evaluation criteria of contradictions, congruences and collaboration is established. Evidence is gathered from participant interviews and the framework is used to surface learning and development obstacles and issues within an expansive learning cycle. The study argues that analysis via this modified AT framework brings original insight. Initial findings indicate that there are relatively few learning and development issues associated with the use of agile tools and techniques themselves and that most problems arise at the interface where the “changed” (more agile) delivery teams meet the organisation’s behavioural norms and practices.

## Introduction

Understanding the difficulties and issues associated with agile implementations has been problematic [8] with many varied perspectives [26], organisational settings and approaches [18]. Previous studies [11] have highlighted the need to consider environmental, behavioural and cultural dimensions when studying software development and a recent study [10] suggested an organisational learning perspective with Activity Theory as a useful lens for examining these elements when implementing and adapting agile delivery practices. This paper adds to the discussion by applying an Activity Theory (AT) based framework to evaluate organisational learning, cultural problems and issues when implementing and adapting agile practices. It addresses the following research questions:

*RQ1: * How can Activity Theory provide a structured framework to understanding learning & development issues when implementing an agile approach?
*RQ2: * What insights does AT give into the learning & development issues that predominate when an organisation transitions to an agile mode of delivery.

An Activity Theory based framework is applied to a large case study organisation text implementing an agile approach and the focus is Engestrom’s notion of expansive learning whereby learning and development within organisations progresses by resolution of contradictions and frictions [13]. Consequently, the development and successful take-up of agile practices will only occur as the organisation progresses through a sequence of identification, consideration and subsequent resolution of multiple contradictions. This paper posits that the identification of these contradictions and their approaches to resolution within an expansive learning cycle provides a useful structured framework that facilitates an original insight into the obstacles and issues that impact agile implementations.

To achieve this objective, this paper defines an activity within an Activity Theory context and then hierarchically deconstructs agile development activities from Agile Manifesto principles to propose a set of six key activities that encompass agile delivery activity. This framework is used to examine the issues that an organisation encounters as it adopted agile delivery practices. This study uses the identification of contradictions, their types and occurrences as well as their resolution and collaborative activity as a structured and progressive indicator of the nature and type of learning and development issues that organisations face in implementing agile approaches. This paper is organized as follows. Section 2 develops an Activity Theory based framework of six generic agile activities. Section 3 outlines the case study organisation and the research method adopted. Section 4 details the study findings in terms of identified contradictions, congruences and collaborative interactions that take place within the agile activities. Section 5 discusses the results and concludes the paper.

## Background and Related Work

Originating within the Cultural-Historical Analytical Theory (CHAT) domain, Activity Theory (AT) provides a framework to examine many aspects of work activity and especially highlights frictions and tensions when new initiatives are developed. Chita [10] provides a fuller account of the learning cycle within an agile development environment. From a learning perspective, Activity Theory helps to focus on the important influence of the environmental mix such as culture, procedures, roles, peers, policies and artifacts.

### Activity Theory Based Framework

**Fig. 1.**
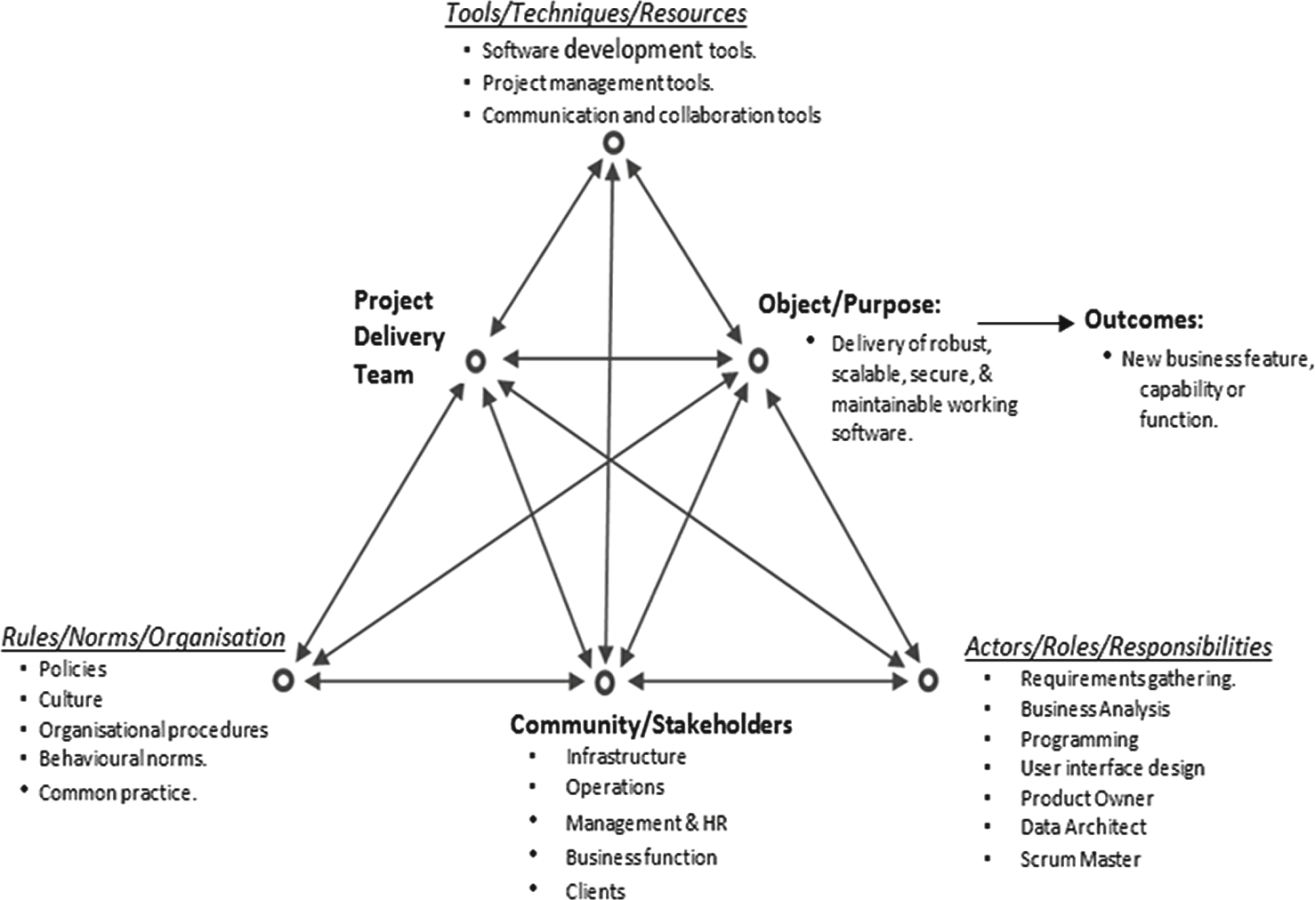
Example Project Delivery Activity (after Engestrom, [13])

Engestrom [13] sees the unit of analysis as collective rather than individual activity [25] and argues that the collective perspective is a useful tool for studying organisational change and learning. Engestrom’s approach is illustrated in Fig. 1. Generic delivery activity of a project team is shown with the focus or purpose of the activity, represented by the horizontal line through the middle of the triangle from the Project Delivery Team node (also known as Subject node) to the Object/Purpose node.

This delivery activity both mediates and is mediated (affected/influenced) by the node representing Tools/Techniques/Resources (e.g. a story board or a work package) which might be used as part of the activity, as well as by the Community/Stakeholders (e.g. clients or management) context node within which the delivery activity takes place.

The relationship between the Project Delivery Team node and the Community/Stakeholders node is mediated by the Rules/Norms/Organisation node and also the relationship between the Community/Stakeholders node and the Object/Purpose is mediated by the Actor/Roles/Responsibilities node that reflects how work and responsibilities are divided and allocated. According to Engestrom [15] there will be contradictions and friction within and between these nodes and also between discrete organisational activities. Allen et al. [1] taking a holistic activity system perspective, define “contradiction” as anything that opposes the overall motive of the activity and the individual or collective aims that the subjects (activity actors) are striving for. These contradictions occur in a progression as the activity evolves and changes through an expansive learning process which occurs as a series of progressive series of contradictions are resolved. The cycle starts with *Primary Contradiction* that emerges as an initial trigger point from within one of the above six nodes. A *Secondary Contradiction* leads to a deeper analysis by the subjects (activity actors) with more detailed questioning and is likely to emerge between two nodes. A *Tertiary Contradiction* emerges as the now evolved or changed activity clashes with the older more established mode of operation [30]. Finally, a *Quaternary Contradiction* occurs when the newly organised or more advanced activity comes up against other organisational activities which are still expecting the interaction to be with the previous older version of the activity.

The above delivery activity represents the overall framework adopted by this study where the focus is on the contradictions, frictions and tensions that are associated with the activity. and this study aims to identify and discuss these and their influence on the process of implementing agile delivery approaches.

### Defining an Activity

This approach relates to the Activity Theory principle of Hierarchical Decomposition which significantly impacts on the unit of analysis [30, 35, 37]. The AT literature mostly refers to one or two key articles [13, 14] and as Sannino [37] points out there have been various critiques of Engestrom’s representation of Activity Theory [13] as a conceptual model for the analysis of social practices.

Cash et al. [9] draw extensively upon Bedny and Karwowski [6] and Bedny and Harris [5] in their approach to building a multi-level theory applied to the engineering design process and they ask the pertinent question, “At what scale do distinct design activities and tasks occur and how are the various scales related?” Cash et al. [9] also indicate that in the design field, studies have taken place at different levels and that there are difficulties in pulling together the implications and relationships of these studies. This could also be said to be true of studies in IS/IT where there are extensive articles on methods and processes as well as programming and interface design [8, 32, 38, 39] but little that actually pulls them together into a coherent whole. Cash et al. [9] state that “as with any technical system, the ability to describe behaviours and properties of the system across multiple scales is essential for generating deep scientific understanding,” and borrowing from Bedny and Karwowski [6] they arrive at an Activity → Task → Action decomposition that differs from the conventional Activity Theory structure of Activity → Action → Operation.

**Fig. 2.**
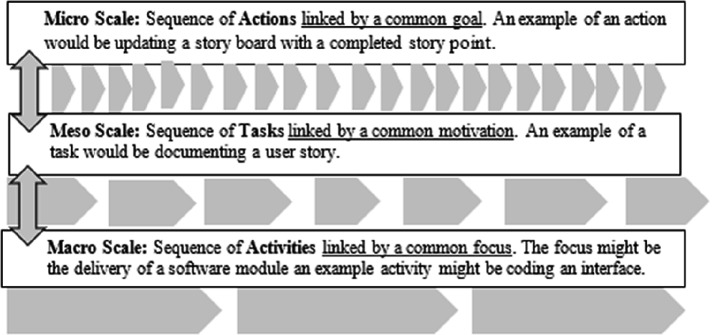
Cash et al. [9] framework adapted for an agile environment.

Bedny and Harris [5] identify the production process as a s*equence* of transformations of raw material into a finished product and Cash et al. [9] apply this to the design process and arrive at an illustrative diagram which has been modified below (Fig. 2) to start at the lowest level (Actions) to arrive at Activities at the highest level and is applied to the agile delivery process.

For gathering research data and analytical purposes, this represents a more granular approach to the application of AT rather than an approach that envisions the whole of the software development process as a single activity. Applying this approach to a traditional delivery lifecycle, one could classify each stage of the cycle as a specific activity. Such a linear approach might well fit some development lifecycles, but most are likely to have more than one activity either taking place simultaneously or across multiple stages.

### Activities in Agile Delivery

Identification of a generic and widely applicable set of agile delivery activities was pursued by re-visiting the twelve agile principles on which much of current agile development activity and practice is founded. What constitutes a “principle” is open to interpretation and general definitions of a principle vary from “a fundamental truth or proposition that serves as the foundation for a system of belief or behaviour or for a chain of reasoning” to “a fundamental source or basis of something” as well as “a general scientific theorem or law” [33].

Meyer [28] posits his own set of principles which he calls a “usable list”, divided into two groups, Organisational and Technical. This consolidates the rationale and core concepts behind the Agile Manifesto Principles into a logical, granular and discrete list. Supporting these principles, Meyer [29] identifies several practices that he regards as the regular “almost ritual” activities that must be undertaken in order to be able to conform with and apply the agile principles. Amending Meyer’s list with additional elements (Requirements Gathering and Learning and Development) we can derive a coherent set of agile activities that cater for the concepts underpinning the Agile Manifesto Principles and which should support all agile delivery approaches, and which can be identified as constituting activities within an AT context. These defined activities are depicted in Table 1 below.

**Table 1.** Generic agile activities and tasks

Agile activity	Description	Example tasks
Development (Dev)	Simple and incremental design; coding standards and shared coding	Deploying coding standards Pair programming; Refactoring
Release Management (RM)	Planning, Continuous Integration and Configuration Management	Estimation tasks Frequent/small releases Configuration Management
Testing and Quality (T&Q)	Test driven development and all aspects of assuring software capability	TDD; Unit testing Defect analysis
Requirements Engineering (RE)	Customer focus, gathering and developing user stories, use cases etc.	Customer collaboration. Manage changing requirements. User story development
Learning and Development (L&D)	Retrospectives for incremental improvement	Sprint retrospectives; Training Project retrospectives
Governance and Support (G&S)	Incorporates Meyer’s management practices and other organisational support elements	Daily stand-ups Self-organised/teams Development environment

This identified set of activities was assembled in a draft paper and circulated amongst senior Agile coaches and consultants within the financial services, public sector and consulting domains in the Edinburgh (Scotland, United Kingdom) area and the feedback to date has been positive and in general agreement with the set.

Having arrived at a defined set of activities and tasks, the initial requirement is to evaluate these suggested activities according to published literature to provide a wider appraisal and comparison with other perspectives that address agile delivery activities and practices. These perspectives are most likely to be addressed in agile maturity model literature, as it aims to align agile processes^1^ and practices/activities either within the traditional maturity model approaches [26] or those that define separate agile maturity models [18]. A recent study by Fontana et al. [17] aims to evaluate “currently proposed agile maturity models” and the authors identified 14 papers that were considered important for their analysis.

This study adopted these same 14 articles as representative of the wider context and perspectives of all elements that make up agile activities and practices and compares them with the proposed set of generic agile activities in Table 1 above. The generic activities were found to map well to all indications of agile activities and practices mentioned within the 14 articles. Given the wide variety of likely organisational situations, and an Activity Theory based approach that progresses bottom up from Actions → Tasks → Activity to arrive at an activity that consists of a number of conceptually linked tasks with a common focus, these agile activities represent a logical, distinct and comprehensive set. Within each of these agile activities and tasks, expansive learning will occur as organisations and delivery teams face contradictions and obstacles within these activities/tasks and either adopt additional practices to resolve them or perhaps pursue them in different ways. Therefore, each organisation will have its own view and/or hybridized version of tasks and artifacts within each of the above six activities.

### Congruences and Collaborative Activity

In their analysis of technology-mediated organisational change, Allen et al. [1] introduce the concept of “congruences” which they see as “temporary stabilization” or stabilizing forces within an activity system, which they regard as a development that leads to balance rather than precipitating change. In this analysis, it is important to recognize elements that promote reproduction as well as those that give rise to change as that whilst there will be tensions that provoke change, there are also issues related to the development of congruences.

Through their analysis of primary and secondary contradictions, Allen et al. [1] argue that these sources of tension give rise to an advanced form of the activity as a result of greater congruencies within the work activity. Their analysis of case studies revealed contradictions being offset by congruencies and through a process of feedback and action, the contradictions were transformed into congruencies [1]. Dennehy and Conboy [12] take up this point and note that the congruence of contradictions within and between activities will act as drivers of change giving rise to several levels of congruency between the different elements. The authors quote Allen et al. [2] who indicate that these congruencies can be immediate where things work better within an activity or give rise to longer term congruencies. The authors [12] argue that it is the congruence of contradictions that is important in explaining the evolution and development of an activity.

Hasan and Banna [20] indicate that innovation and the resolution of a contradiction has to take place at the social level and cannot happen at the individual level. They point to Bodker’s [7] work in HCI who indicates that there has to be close collaboration and cooperation to deliver better design. Engestrom [15] and Bardram [3] have also considered this element in their examination of collaborative activity. Engestrom et al. [16] identified a progression of three levels of collaborative activity taking place.

Co-ordination is the “normal scripted flow of interaction” [16, p. 372] where individuals will focus on their own assigned roles, objects and actions. The script may consist of written rules and unwritten traditions and participants within the activity are coordinated without question or discussion. In the context of a software development environment, Barthelmess and Anderson [4] indicate that there is a lack of a community concept in this type of collaborative activity and it may be noted that in the context of organisational process or practice this level of activity might be that which is typically incorporated into a traditional maturity model perspective. Progression to the co-operation level of collaborative activity, involves actors that will instead of focusing on their assigned roles will focus on a shared problem or object in order to find an agreed solution. Actors (subjects) will move beyond the confines of a script but will not explicitly question or reinterpret it. According to Bardram [3] the important difference between coordinated and co-operative work is a shared objective and the actors have to balance their own actions with those of their activity partners to achieve a common goal.

Finally, to achieve the co-construction level of collaborative activity, actors will reconceptualize their roles and interactions with the shared object or problem. According to Bardram [3] the objective (motive) of the work is not stable and has to be collectively constructed which he calls “co-construction”. According to Engestrom et al. [16] the script may be re-conceptualised as well as the individual’s interactions with each other. Actors will pose questions such as “What is the meaning of this problem in the first place? Why are we trying to solve it - and who benefits from its solution? How did the problem emerge?” [3: 9].

Barthelmess and Anderson [4] indicate that there is a close interplay between these different levels as they are all part of “collaborative activity” and that a pattern of dynamic transformations between these levels can be observed. To illustrate using a software development example, writing software might occur in a coordinated way and a developer might encounter a problem perhaps with a specification or a tool (contradiction). This might then become a collaborative activity as the developer and business analyst collaborate with regards to problem resolution and once resolved activity returns to a coordinated state. Alternatively, it might be a serious problem that requires a more considered approach that involves re-thinking practice in which case the activity becomes a co-constructive effort at which point practice is questioned and re-conceptualized and expansive learning takes place as contradictions are resolved. The activity then returns to the coordinated state.

In the case study organisation below, the different types and details of contradictions within the agile implementation are identified in order to determine the learning and development issues that the organisation encounters when implementing an agile approach. The occurrence of different types of collaborative activity as pre-cursors to expansive learning are also examined.

## Case Organisation and Study Design

This research focuses on a single case study organisation with the intention to identify the nature and type of contradictions, congruences and collaborative activity that has occurred within the organisation during its adoption of the Structured Agile Framework (SAFe) method. According to Runeson and Host [36] the case study methodology is well suited to software engineering research and provides a deeper understanding of the phenomena under study. The case study organisation is a large public sector body that delivers a broad range of services. As a single body employing over 3500 people, the organisation leverages efficiencies of scale and reduced infrastructure costs in the delivery of its services for which the demand has grown rapidly creating many challenges which led to the creation of the Change Programme which ran from May 2017 to April 2019.

The Programme was initiated by senior management and the IT and Programme Support functions decided to adopt and use agile approaches in a very short space of time leading to the rapid deployment of the SAFe framework. This represented a major change from the waterfall and PRINCE2 based approaches previously deployed. Given the starting point, scale, speed of implementation and the requirement to urgently deliver value in the complex public sector environment, the Change Programme had many significant learning and development issues. Over the two-year duration of the Change Programme, a core group of around 100 people were involved but intermittently this grew to nearly 200 people, divided into twelve delivery streams.

This paper presents the initial results from an analysis of semi-structured interviews conducted so far with 13 delivery managers involved in the programme. The hour-long interviews were with senior managers responsible for delivery streams and took place in the period immediately after the programme ended in April through to September 2019. The interview questions were derived from a series of previous papers that have applied Activity Theory to case study organisations [21–23, 27, 31, 34]. Thirty-seven interview questions were derived and were designed to be as widely applicable as possible. In addition, illustrative diagrams were used to guide the interviewees. The NVivo (v12) qualitative data analysis tool was used to code the interview transcripts for the occurrences of contradictions, congruences and collaborative activity. Interview transcripts were also examined for statements indicating problems & issues related to the Change Programme’s agile approach.

The interviews were conducted immediately after the Change Programme had finished and so individual’s perspectives were current and relevant. Interviewees were forthright and open in their responses and were keen to divulge their views and perspectives. As the interviews were conducted at the delivery manager level, the results are likely to reflect broader issues that concern delivery managers rather than immediate software development and build issues. Consequently, identified contradictions and congruences such as those relating to Governance & Support, Learning & Development activities are likely to predominate. With the programme having terminated and with over 100 core personnel involved having returned to their core functions there was little opportunity to engage in observational research to enable the collected data to be triangulated.

## Findings

The findings are structured into three sections related to contradictions, congruences and indications of collaborative interactions. For contradictions, the type and levels were identified and for congruences only levels could be established. For collaborative interactions, attention focused on instances of co-operation and co-construction.

### Contradictions

Instances of the four types of contradictions across all six generic agile activities are depicted in Table 2 below; the last column indicates the number of mentions and discussions of discrete elements within the interview transcripts.

**Table 2.** Contradiction frequency

Contradiction	Description	Mentions
Primary	Occur within the six nodes	51
Secondary	Occur between the six nodes	318
Tertiary	Occur between the activity and an advanced form	20
Quaternary	Occur between the activity and neighbouring activities	53

The nodes in Table 2 relate to the six points of the Activity Triangle in Fig. 1 and the “advanced form” relates to an improved version of the activity. There are few initial tensions and contradictions within individual nodes as indicated by the relatively few *Primary Contradictions.* This indicates that individuals do not experience many issues or tensions or difficulties within nodes such as the delivery teams or the tools and techniques per se used when implementing agile methods. The large number of *Secondary Contradictions* indicate by a significant margin that most of the frictions and tensions occur between nodes as the delivery activities evolve and more questions are being asked. A summary of these secondary contradictions is displayed below in Table 3.

**Table 3.** Secondary contradictions analysis

Secondary contradiction	Example description	No.
Subject – Artefact	Use of agile tools and techniques by delivery team	77
Subject – Rules & Norms	Delivery team practices and norms	19
Subject – Div. of Labour	Allocation of roles and work within delivery team	20
Community – Artefact	Use of agile tools and techniques by other stakeholders with an interest in the activity	24
Community – Rules & Norms	Organisation wide practices and norms	150
Community – Div. of Labour	Division of labour within the other stakeholders	14

Within the project delivery teams most issues revolved around the use of the agile tools and approaches (77). Typical issues related to the understanding and deployment of agile techniques and the mixed level of training that was provided as was illustrated by one delivery stream manager.

*“I think it would have been better if I had been trained and knew how the organisation wanted to implement it. But I had other people, like I know that other people received really good support and back-up”*

To a much lesser extent, the delivery team came up against issues regards adopting agile practice and norms compared to existing team delivery practices as illustrated by another delivery stream manager.

*“we had real difficulties because solution architects their job and title is thinking about solutions. But of course, when you are running in an agile way you are kind of solution agnostic until the point you have gathered all your engineering requirements”*

By far the most prevalent secondary contradictions (150) occurred beyond the delivery team, within the area involving the wider organisational groups who had a vested interest in the delivery activity and who interfaced with the activity in terms of the organisational rules, procedures and normal practices that were deployed. The magnitude of the issue is illustrated by one delivery stream manager.

*“Very simply we work in an organisation of three and a half thousand people and there was only 200 people on an agile programme so we’re not, we’re not going to change the way those two or two strands run in the organisation for 200 people.*

The existing organisational structure continued to pose issues throughout for the whole programme There were few *Tertiary Contradictions* (20) which indicates that either there were not many tensions and frictions with moving to a more evolved version of the agile activities and an overall willing preparedness to embrace newer approaches. Alternatively, it could mean that the agile activities are not yet evolved to a point that demanded the older ways needed to be abandoned. Typically, the main difficulty centered around individuals reverting to previous ways of working as was mentioned by the programme director.

*“And then what happens is that, if you get people joining a team, they don’t get the proper training, and then if they’ve got five things to manage, it’s easier for them to default to their existing ways of working. So, I think that’s been an issue with […], and I would say generally an issue with SAFe and Scrum, is if you don’t have dedicated resources, it’s really hard to make it stick, because people just get pulled back into, you know…if the environment doesn’t change, you get pulled back into the same ways of working”*

There were far more *Quaternary Contradictions* (53) that occurred, and one delivery stream manager put it rather tersely: *“We clashed with probably every part of the organization”.* Others indicated the repetitive nature of continually having to engage with and educate multiple organisational elements.

“*it’s harder to control because you’re bringing in business units and they’ve got their old ways of working and they’re not necessarily motivated because they’ve not been in the programme for a year and getting used to ways of Agile and all that kind of thing. And so, you felt that you were having to start again, and then again in the next increment, and again as soon as another service came on”*

From the above it is apparent that different people that are engaged in different activities are facing a variety of learning and development issues all at various stages of the expansive learning cycle as the programme engages with a different way of working. The least problematic area is overcoming the reluctance of individuals and organisational units to let go of older approaches (*Tertiary*). The introduction and use of new tools and techniques (*Primary*) and the interface that an evolved new activity (*Quaternary*) has with the rest of the organisation is slightly more problematic but by far most of the tensions and frictions and therefore learning and development opportunities relate to where the change programme’s developing and evolving activities interface with the rest of the organisation’s existing norms and practices (*Secondary*).

**Table 4.** Generic agile activities and contradictions frequency

Generic agile activity	No.
Governance and Support (G&S)	80
Release Management (RM)l	40
Learning and Development (L&D)	57
Requirements Engineering (RM)	14
Testing and Quality (T&Q)	2
Building and Coding (B&C)	4
**Stream** – contradictions affecting whole stream	108
**Programme -** contradictions affecting whole programme	179

Of these evolving and developing activities the occurrences of tensions and frictions is not evenly distributed across all the generic activities as illustrated in Table 4 above. This shows programme wide contradictions dominate followed by those affecting a single stream and then those that affect the Governance and Support (G&S) generic agile activity. However, this may well reflect the perspectives of the senior management individuals interviewed so far. This would also explain why Building and Coding (B&C) and Testing and Quality (T&C) are so low.

### Congruences and Stabilizations

The following table details the occurrences of Congruences and Stabilizations within the analysis conducted so far (Table 5).

**Table 5.** Congruences and stabilizations occurrences

Congruences and stabilizations	No.
Primary – within a node	21
Secondary – between nodes	61
Tertiary – between an activity and an advanced version	11
Quaternary – between activity and an adjacent activity	34

As can be seen the most common occurrences of congruences are the congruences that relate to secondary contradictions. Given that secondary contradictions emerged as the most common in the previous analysis then the higher number of secondary congruences is indicative of substantial efforts to address the contradictions. The following quote from a delivery stream manager is indicative.

*“All of the scrum event planning, retros, reviews and scrum they help support the team. We knew we had to go to those we did go to those, they supported us, they allowed openness so the events themselves worked very well for us. Helped knowledge management and sharing. In relation to capacity and estimation that was really, really good because for the first time probably we weren’t just assuming that everybody was there all the time”*

### Collaborative Activity

Co-ordination activities are prevalent all the time and have not been identified as they are not indicative of any progression towards expansive learning activity.

**Table 6.** Collaborative activity

Congruences and stabilizations	No.
Co-ordination – not looked for	N/A
Co-operation	36
Co-construction	6

Table 6 provides a high-level perspective indicating the presence of substantial levels of co-operative activity which is a significant pre-cursor to expansive learning taking place [16]. There were many illustrative examples of this such as the following:

*“So, what I think it did is, the hand raisers found themselves in it and what it did is it raised an awareness of what was possible, let’s look, here’s a way of working. And I was one of them, I didn’t raise my hand to be in it but I found myself or the universe found me in it, and I think it raised a kind of oh this is what’s possible, this is really exciting*.”

Examples of co-construction were very limited but there were indicators of a supportive environment that would facilitate such activity.

*“I think all my people, regardless of age or inclination, are probably full of good ideas, but they all require different ways of getting those ideas to come out of their mouths, so Agile will help some of them”*

Identifying examples of collaborative activity simply shows at a very high level the propensity or potential of the individuals and organisational units to make progress along the expansive learning cycle. With the interview transcripts analysed so far, it’s not been possible to link collaborative activity to the different levels of contradictions and congruences, but this is a later aim of this study.

## Discussion and Conclusion

Focusing on the learning and development aspects, this study proposes an alternative structured approach that is granular and progressive, and which helps to identify and understand the issues that an organisation encounters when implementing agile. When they examined the challenges facing organisations implementing agile, Gregory et al. [19] identified seven major themes and twenty-seven sub-themes in the data collected. The highly diverse themes ranged from organisational elements to cultural aspects to sustainability elements to business value. Whilst a very useful list of elements there is little likelihood of identifying inter-relationships or connective elements perhaps precisely due to the wide diversity of the issues identified.

The analytical approach taken in the paper of viewing the issues identified within an Activity Theory framework has been shown to provide an inter-connected context which places these issues in a useful progressive framework. For example, anything to do with teams whether it is team practices or recruitment relates to the subject node and issues with teams themselves constitutes a primary contradiction. Team practices using new approaches constitute a secondary contradiction. Organisational culture elements and business value aspects can relate to Rules & Norms node and Distributed Teams relates to the Division of Labour nodes. Primary contradictions will relate to issues within teams and once they are resolved then attention will turn to secondary contradictions that occur beyond the subject or team nodes. This offers a form of a progression of issues and the value of the Activity Theory framework is that it places these issues within a structure where resolution of contradictions & congruences leads onto the next step in the Expansive Learning Cycle. This Activity Theory based framework identifies contradictions at particular levels to provide a useful insight and understanding in terms of locating where the major issues are in a progressive cycle compared to simply identifying a list of different types of issues that the organisation faces without any context of importance or contribution regards progress towards an organisation successfully transitioning to an agile mode of delivery.

As evidenced by the number of occurrences of *Primary Contradictions,* this study indicates that in this case, most issues do not relate to the actual use of agile tools and techniques or even a reluctance to let go of previous ways of working as evidenced by the relatively few occurrences of *Tertiary Contradictions*. The location of these contradictions within the six generic agile activities facilitates a more structured and granular approach to locating specific issues. In this study the contradictions have been mostly concentrated around the Governance & Support tasks, though this is likely to reflect the focus of the delivery managers interviewed. As indicated by the large number of *Secondary Contradictions*, the major tensions and frictions relate to the interface between the delivery team and the rest of the organisation and its practices and behavioural norms. This is consistent with the findings of other authors such as Gregory et al. [19] and Kuusinen et al. [24]. These issues extended beyond specific agile activities and most affected the whole programme and many related to individual delivery streams.

The findings indicate that the agile activities that experienced substantial issues were related to Governance and Support (G&S) as well as Learning and Development (L&D) activities. The G&S activity would be expected to be significant due to the large number of contradictions identified that related to the interface between the delivery team and the rest of the organisation. It could also be indicative of the management level of individuals who have been interviewed so far. The L&D contradictions seem to be derived partially from the mixed levels of formal training and development that was made available to the participants although there is also evidence of substantial provision of mentoring and support provided throughout. This presents an area for further analysis within this study, for instance examining the reasons the organisation is less willing to fund formal training but is willing to spend on mentoring and coaching and whether this relates to budget holders or funding cycles.

The distribution of congruences broadly follows that of the contradictions which is indicative of significant attempts to address and resolve the occurring contradictions. The occurrence of collaborative interactions particularly in terms of co-construction is extremely limited and is perhaps indicative of the limited opportunity for individuals to reconceptualize their roles and interactions. With regards to the second research question, this approach could indicate where learning and development issues predominate when implementing agile an agile approach. The findings point to specific areas for further research, particularly in the area of the impact of organisational practices and norms as well as individual attitudes and autonomy. This study is confined to delivery managers and further research could consider interviewing delivery personnel as well obtaining perspectives from business units benefiting from the change initiative as well as business functions supporting the initiative such as finance and HR.

This study has provided a framework to map defined Activity Theory concepts to agile delivery processes. It has used the concepts of contradictions, congruences and collaborative interactions to suggest a structured framework to view obstacles to learning & development encountered by organisation. With regards to the first research question this structured, granular, generic and scalable approach provides a framework that moves beyond a checklist approach of issue identification and the study findings should complement existing approaches of both academics & practitioners as they examine the issues and difficulties of implementing agile delivery methods.

## Footnotes

1 This study views a process as a sequence of activities/practices.

## References

[1] Allen DK, Brown A, Karanasios S, Norman A (2013). How should technology-mediated organizational change be explained? A comparison of the contributions of critical realism and activity theory. MIS Q..

[2] Allen DK, Karanasios S, Norman A (2014). Information sharing and interoperability: the case of major incident management. Eur. J. Inf. Syst..

[3] Bardram, J.: Designing for the dynamics of cooperative work activities. In: Proceedings of the 1998 ACA4 Conference on Computer Supported Cooperative Work, Seattle Washington (1998)

[4] Barthelmess P, Anderson KM (2002). A view of software development environments based on activity theory. Comput. Support. Coop. Work.

[5] Bedny GZ, Harris SR (2005). The systemic-structural theory of activity: applications to the study of human work mind. Cult. Act..

[6] Bedny GZ, Karwowski W (2004). Activity theory as a basis for the study of work. Ergonomics.

[7] Bodker, S.: Activity theory as a challenge to systems design. in information system research: contemporary approaches and emergent traditions. In: Sanstrom, G., Nissen, H.E. (eds.) Proceedings of the IFIP TC 8/WG 8.2 Working Conference. Elsevier

[8] Boehm B, Turner R (2005). Management challenges in implementing agile processes in traditional development organisations. IEEE Softw..

[9] Cash P, Hicks B, Culley S (2015). Activity theory as a means of multi-scale analysis of the engineering design process a protocol study of design in practice. Des. Stud..

[10] Chita, P.S.: Agile Software Development – Adoption & Maturity. in Agile Processes in Software Engineering and Extreme Programming. XP2018

[11] Dennehy D, Conboy K (2017). Going with the flow: an activity theory analysis of flow techniques in software development. J. Syst. Softw..

[12] Dennehy D, Conboy K (2019). Breaking the flow: a study of contradictions in information systems development (ISD). Inf. Technol. People.

[13] Engestrom Y (1987). Learning by Expanding: An Activity-Theoretical Approach to Developmental Research.

[14] Engestrom Y (2000). Activity theory as a framework for analyzing and redesigning work. Ergonomics.

[15] Engestrom Y (2001). Expansive learning at work: towards an activity theoretical reconceptualization. J. Educ. Work.

[16] Engestrom, Y., Brown, K., Christopher, L.C., Gregory, J.: Co-ordination, cooperation and communication in the courts: expansive transitions in legal work. In: Mind, Culture and Activity: Seminal papers from the Laboratory of Comparative Human Cognition, p. 239 (1997)

[17] Fontana RM, Albuquerque R, Luz R, Moises AC, Malucelli A, Reinehr S, Larrucea X, Santamaria I, O’Connor Rory V, Messnarz R (2018). Maturity models for agile software development: what are they?. Systems, Software and Services Process Improvement.

[18] Fontana RM, Fontana IM, Garbuio PA, Reinehr S, Malucelli A (2014). Processes versus people: how should agile software development maturity be defined?. J. Syst. Softw..

[19] Gregory P, Barroca L, Sharp H, Deshpande A, Taylor K (2016). The challenges that challenge: Engaging with agile practitioners’ concerns. Inf. Softw. Technol..

[20] Hasan, H., Banna, S.: The unit of analysis in IS theory: the case for activity. In: The Fifth Biennial ANU Workshop on Information Systems Foundations. pp. 1–8. ANU, Canberra (2010)

[21] Jonassen DH, Rohrer-Murphy L (1999). Activity theory as a framework for designing constructivist learning environments. Educ. Technol. Res. Dev..

[22] Kaptelinin, V., Nardi, B., Macaulay, C.: The Activity Checklist: A Tool for Representing the “Space” of context. Interactions July/August (1999)

[23] Korpela, M., Activity Analysis and Development in a nutshell. Handout Version 2 (1999)

[24] Kuusinen, K., Gregory, P., Sharp, H., Barroca, L.: Strategies for doing agile in a non-agile environment. In: Proceedings of the 10th ACM/IEEE International Symposium on Empirical Software Engineering and Measurement, pp. 1–6 (2016)

[25] Leonťev AN (1978). Activity, Consciousness and Personality.

[26] Maier AM, Moultrie J, Clarkson PJ (2012). Assessing organizational capabilities: reviewing and guiding the development of maturity grids. IEEE Trans. Eng. Manage..

[27] Martins, L.E.G., Daltrani, B.M.: An approach to software requirements elicitation using precepts from activity theory. In: 14 IEEE International Conference on Automated Software Engineering, pp. 15–23 (1999)

[28] Meyer B (2014). Agile!: The Good, the Hype and the Ugly.

[29] Meyer, B.: Agile Software Development. Online EDX course. https://www.edx.org/course/agile-software-development. Accessed 18 Sept 2018

[30] Mursu A, Luukkonen I, Toivanen M, Korpela M (2007). Activity theory in information systems research and practice: theoretical underpinnings for an information systems development model. Inf. Res. Inte. Electron. J..

[31] Mwanza, D.: Where theory meets practice: a case for an activity theory based methodology to guide computer systems design. In: Proceedings of Interact 2001: Eighth IFIP TC 13 Conference on Human-Computer Interaction, Tokyo, Japan (2001)

[32] Newell S, Galliers RD (2006). Facilitating – or inhibiting – knowing in practice. Eur. J. Inf. Syst..

[33] Oxford dictionaries. https://en.oxforddictionaries.com/definition/principle

[34] Quek A, Shah H (2004). A comparative survey of activity-based methods for information systems development. ICEIS.

[35] Roth WM, Sannino A, Daniels H, Gutierrez K (2009). On the inclusion of emotion, identity and ethico-moral dimensions of actions. Learning and Expanding with Activity Theory.

[36] Runeson P, Host M (2009). Guidelines for conducting and reporting case study research in software engineering. Empir. Softw. Eng..

[37] Sannino A (2011). Activity theory as an activist and interventionist theory. Theory Psychol..

[38] Sauer C, Horner BH (2009). Rethinking IT project management: evidence of a new mindset and its implications. Int. J. Project Manage..

[39] Uden L, Valderas P, Pastor O (2008). An activity theory based model to analyse web application requirements. Inf. Res..

